# Is there a role for higher cognitive processes in the development of obesity in humans?

**DOI:** 10.1098/rstb.2022.0208

**Published:** 2023-09-11

**Authors:** Suzanne Higgs

**Affiliations:** School of Psychology, University of Birmingham, Edgbaston, Birmingham B152TT, UK

**Keywords:** eating, cognition, causes of obesity, memory, inhibitory control

## Abstract

Cognition underpins the flexibility of human eating and disruption to higher cognitive processes, such as inhibitory control and memory, and can result in increased food intake, which in the long term could result in weight gain. The aim of this review is to provide an overview of the current evidence on cognition as a causal factor in the development of obesity in humans. Evidence from meta-analyses supports the suggestion that cognitive function is cross-sectionally associated with obesity even when controlling for a range of confounding variables. However, this association could be explained by reverse causality because there is also evidence that the metabolic syndrome and a history of excess western diet consumption alters brain structure and cognitive function. Data from longitudinal and interventional studies and from non-human animal models suggest a reciprocal relationship between obesity and cognitive function exists but whether disruption to higher cognitive processes is a primary cause of obesity in humans remains unclear.

This article is part of a discussion meeting issue ‘Causes of obesity: theories, conjectures and evidence (Part I)’.

## 1. Introduction

Obesity is a prevalent disease characterized by an excess amount of body fat that impairs health [1]. In broad terms, obesity arises from the interaction between genetic and environmental factors that act through behaviour and underpinning psychobiological processes to alter energy intake and/or expenditure. Research on the specific psychobiological processes that may contribute to the development of obesity has burgeoned in recent years and it has been argued that disruption to higher cognitive processes contributes to overeating and weight gain [2–4]. The aim of this review is to draw together the recent literature (focusing on studies of humans) and to evaluate the evidence in relation to the claim that higher cognitive functions play a causal role in the development of obesity.

## 2. Why might higher cognitive functions constitute a causal factor in the development of obesity?

Higher cognitive functions are mental processes that allow the organization, control and flexible adaptation of behaviour. These functions include processes that come under the umbrella of executive function: inhibitory control, working memory and cognitive flexibility [5], as well as episodic memory (memory for specific personal events) [6]. Human eating is a complex behaviour that is notable for its flexible adaptation to internal and external factors. We may experience feelings of hunger and seek out food on one occasion but on another occasion put off eating and prioritize other activities. We might be drawn to a high-calorie tasty food choice but decline consumption if we have recently eaten or decide to opt for a lower calorie option if we have a goal to reduce consumption of certain foods for health. Higher cognitive functions underpin this flexibility and so disruption of such processes could result in eating that that is less sensitive to moderating influences and/or more driven by automatic responses to food [7]. In the context of an environment characterized by an abundance of high calories foods, such behaviour could result in high levels of consumption leading to weight gain. Disruption to higher cognitive processes could also affect the propensity to engage in physical activity patterns and so reduce energy expenditure [8], but given that changes in the food environment have been identified as the likely primary driver of the recent population increase in the prevalence of obesity [9], the focus of this review is on the link between cognition, energy intake and obesity.

## 3. Higher cognitive functions and flexible eating

The inhibition of an impulse to consume one food item (e.g. fries) over another (e.g. salad) owing to having a healthy eating plan is an example of flexible eating. The ability to resist the tasty fries and align behaviour with a longer-term goal is often referred to as self-control. Key to this conceptualization is that self-control (or will-power) involves resolving a conflict between competing goals: on the one hand, the immediate desire to consume a tasty food and on the other hand, a health goal to avoid consumption of high-calorie foods. According to dual process theories, enacting self-control relies on a reflective/flexible system in the brain that encodes long-term goals suppressing an impulsive/automatic system that encodes immediate rewards [10]. Disruption to the neural processes that underlie this ability to inhibit automatic responding to calorific foods may result in positive energy balance. Supporting the dual process view, there is evidence that tasty food cues can elicit strong conditioned responses including craving and automatic approach tendencies (reflected in enhanced activity in reward-related brain regions) but that with effort, these responses can be suppressed (which is reflected in activity in control regions such as the dorso-lateral prefrontal cortex (dlPFC)) [11,12]. Conversely, experimental attenuation of dlPFC activity using repetitive transcranial magnetic stimulation increased intake of palatable food via stimulation-inductions reductions in inhibitory control [3].

Although inhibition of automatic responses applies to some aspects of dietary decision making, it does not capture the full complexity of the choices that we make about whether, what, when, where and how much to eat. Consider the following: you decide to get a coffee with a friend and your friend takes a cake with their drink. In this situation you may forgo having the cake, not because you are resisting a temptation, but because in that moment the cake is not an attractive option. There may be several factors that weigh into this decision such as the fact that you have not long had your lunch, the cake is expensive, and it is not your favourite flavour. This kind of situation is better accounted for by value-based choice models of self-control which suggest that deciding between two options (e.g. take cake or not take cake) involves a cost benefit evaluation of the attributes and consequences of enacting each choice, with the most valuable action in that moment wining out [13]. Importantly, in the case of eating-related decisions, this kind of framework provides one account of how nutritional state can influence consumption behaviours, since homeostatic signals can be integrated alongside other inputs such as taste and monetary costs to affect the current reward value assigned to a food choice [14]. Accordingly, food choices leading to weight gain would not be viewed as failures of self-control but merely the outcome of the process of integrating multiple factors that influence the attractiveness of the choice.

Cognition is involved in the construction of value-based choices. For example, working memory is required for the integration of decision inputs and working memory capacity will affect the number of inputs/options that are considered [15]. Episodic memory is involved in predicting the value of outcomes/attributes based on past experiences [6]. Therefore, disruption to these processes could result in biased choices that favour overconsumption and weight gain, especially in an environment in which attractive foods are readily available. One example of biased choice would be failing to attend to the predicted effect of consumption on the body resulting in reduced sensitivity to satiety signals. Another would be undervaluing the delayed benefits of a choice (known as delayed discounting) which favours immediate enjoyment of consuming a food over future effects on health. Various lines of evidence support the role of working and episodic memory in food choices, including that there is a positive correlation between working memory and fruit/vegetable intake [16,17] and that the relationship between successful dietary restraint and healthy food intake is mediated by working memory capacity [18,19]. There is also evidence that disruption to episodic memory impairs satiety [20,21]. These data highlight the importance of higher cognitive functions in food choices but is there evidence that disruption to cognition in results in weight gain and the development of obesity?

## 4. Cross-sectional associations between higher cognitive functions and obesity in humans

Several studies have examined the cross-sectional association between Body Mass Index (BMI) and cognitive function in adulthood. Meta-analyses of the results of these studies suggest that higher BMI is associated with lower performance on tasks assessing working memory, inhibitory control and delay discounting [22–25]. Similar associations have been reported for children and adolescents [26] with the strongest evidence supporting an association between poor inhibitory control and higher BMI [27,28]. There have been fewer studies that have assessed the relationship between obesity and episodic memory function, but most have reported a negative association [29]. Cheke *et al.* [30] examined performance on a computerized episodic memory task that required participants to ‘hide’ items around a complex scene and then recall the identity, location and timing of when an item was hidden. Participants with obesity performed less well on spatial, temporal and item memory and made more errors when combining these elements into a ‘what–where–when’ memory. Other aspects of task performance were unaffected (e.g. reaction time), which is consistent with the suggestion that obesity is associated with reduced higher cognitive function specifically rather than a general decline in ability to perform a task [31]. Higher BMI in children has also been associated with poorer performance on a spatial episodic memory task [32]. The association between obesity and cognitive function has further been reported to be robust to adjustment for various confounders including demographics, education and health conditions [33,34] but few studies have controlled for the effects of depression and stigma directed towards people with a high BMI, even though these factors are known to be associated with poorer cognitive function [35,36]. An umbrella review that included a quantitative synthesis of effect sizes from published meta-analyses reported a small but significant relationship between several measures of higher cognitive function and heavier body weight [37].

The results from behavioural studies are supported by the results from studies that have examined brain function in adults with and without obesity using brain imaging techniques. There is evidence that having obesity is associated with altered functional connectivity between brain regions. A meta-analysis of 29 studies that used resting-state functional magnetic resonance imaging found evidence that higher BMI is associated with decreased functional connectivity in networks associated with cognition and increased connectivity in networks associated with reward processing [38,39]. There is also more recent evidence for disruption to the hierarchical functional organization of large-scale networks in the brain in obesity [40]. An analysis of studies that examined neural responses to visual presentation food cues in the scanner found that compared with lean participants, participants with obesity had reduced activation in regions associated with cognitive control and increased activation in reward-related brain regions [41]. A recent meta-analysis found no evidence for reduced activity in cognitive control areas in obesity [42] but this result may be explained by the fact that only passive food viewing paradigms were included in the analysis and studies assessing activation during tasks requiring inhibitory control were excluded.

Obesity is also associated with altered brain morphology. The results from four meta-analyses report an association between higher BMI and reduced grey matter (GM) volume in areas of the brain that underpin higher cognitive function including the prefrontal cortex [43–46]. These effects have been reported to be present from an early age and have been linked to cognitive performance [47,48]. The collective results from individual small-scale studies are supported by findings from big data studies that have analysed findings from large population cohorts (e.g. of data from 40 000 individuals in the UK Biobank database) [49]. Taken together, these data suggest that having obesity is associated with reduced GM volume in areas of the brain associated with higher cognitive functions.

Studies on white matter (WM) microstructure have reported that obesity is associated with reduced WM integrity (e.g. [50]), but the pattern of findings, albeit from a limited number of studies, has been inconsistent. The first meta-analysis of 16 studies found an association between having obesity and reduced WM integrity in the anterior part of the corpus callosum, a tract that links frontal areas involved in higher cognitive functions [51]. A more recently published analysis of 31 studies of adults and children found evidence for altered WM integrity in obesity but an inconsistent pattern across studies [52]. A recent large-scale study of data from the UK Biobank reported no association between measures of central obesity (e.g. waist to hip ratio) and WM volume or integrity [53]. The inconsistencies in results relating to WM tract structure may be explained by the small number of studies conducted to date that have used a range of different measures to assess WM integrity. In addition, control for confounding variables (e.g. age, sex, comorbidities) has been inconsistent across studies. A comparison of WM integrity in adolescents with obesity who either also had or did not have type 2 diabetes, reported changes in WM integrity associated with diabetes, but not with obesity [54]. Having type 2 diabetes is associated with reduced cognitive performance which is mediated by a range of comorbidities including sleep disturbances, micro vascular problems and depression [55]. Controlling for a range of comorbidities is necessary to draw conclusions about the specific association between obesity, brain structure and cognition.

Collectively the data from cross-sectional behavioural and brain imaging studies converge to suggest that obesity is associated with disrupted higher cognitive function, particularly in the domains of inhibitory control and memory. However, the results from these studies do not shed any light on whether the cognitive profile is a causal factor in the development of obesity. In fact, it is possible that no causal relationship exists, and that the association is explained by a common third factor exerting an independent effect on both cognition and obesity. There could be causal pathway from cognition to obesity, but the cross-sectional association could equally be explained by the reverse causal pathway: an impact of adiposity on cognitive function. Indeed, there is evidence that prolonged consumption of a high fat diet, accumulation of excess adipose tissue and development of the metabolic syndrome is associated with reduced cognitive function via several mechanisms including oxidative stress, inflammation, insulin resistance and altered neurochemical signalling [56–59]. Cross-sectional measurement of variables at one time point, means that it is impossible to draw conclusions about whether obesity precedes altered cognitive function or the other way round. Longitudinal studies allow for testing of the temporal relationships between variables, which is necessary to show a cause precedes an effect. Testing over multiple time points at an early age before weight gain has occurred also reduces the possibility that any association between cognition and adiposity is explained by the long-term effects of dietary pattern and/or living with obesity on the brain.

## 5. Longitudinal associations between higher cognitive functions and obesity

Poorer inhibitory control and reduced ability to wait to obtain a higher reward have been linked to weight gain in children (e.g. [60,61]). Children with low executive function were also found to have a significantly higher probability of transitioning to a pattern of behaviour characterized by a high-calorie low-nutrient diet over 30 months than did children with high executive function [62].

Several recent studies have examined predictors of weight gain using data from the Adolescent Brain Cognitive Development (ABCD) Study, which is a longitudinal, observational study of over 10 000 children recruited at ages 9–10 years from 21 sites throughout the United States [63]. Hall *et al.* [64] reported that increased volume, thickness and surface area in a region containing the dlPFC, predicted lower BMI one year later and that this relationship was mediated by performance on a task assessing non-verbal abstract reasoning. Children with obesity (but not lean children) had lower GM volume in areas including orbitofrontal cortex, hippocampus, caudate, amygdala and thalamus at 2-years follow-up compared to baseline [65]. An analysis by Adise *et al.* [66] identified brain structural predictors of belonging to a weight-gaining versus weight-stable group of youth from the ABDC study, including reduced cortical thickness in frontal areas. However, these predictors differed from previously identified predictors of 1-year weight gain in the same cohort [67], which requires further investigation.

The results of longitudinal studies in children are consistent with the suggestion that cognition may have a role to play in the development of obesity, but given that some children in the cohorts studied had already developed obesity it is not possible to draw definitive conclusions about causality. Indeed, there is evidence to support a reciprocal relationship from longitudinal studies whereby obesity predicts cognitive function which in turn predicts greater adiposity. For example, a study of a large cohort of children found that greater adiposity at the age of 9 predicted poorer working memory at the age of 10 but also that poorer working memory at the age of 10 predicted greater adiposity at the age of 15.5 [68]. A meta-analysis of 18 longitudinal studies conducted in children and adolescents found that executive function, particularly inhibitory control, predicted weight status and the development of obesity [69]. The opposite relationship was also found whereby weight status was associated with poorer executive function, particularly working memory [69]. Bidirectional associations between obesity and cognition have also been reported in a large cohort of adults from the Canadian Longitudinal Study on Ageing [70]. Higher baseline waist circumference was associated with lower executive function at 3-year follow-up for middle aged adults and lower baseline executive function predicted higher waist circumference at follow-up. Hence, in longitudinal observational studies where measurement occurs after there has already been a chance for obesity to develop, it is not possible to establish which factor initiated the reciprocal relationship. In addition, although it is possible to control for potential confounding variables, confounding cannot be ruled out in observational studies.

## 6. Strengthening causal inference in observational studies

Causal inference in observational research can be strengthened by using methods that exploit genetic information. These approaches rely on the fact that genetic variation temporally precedes outcomes. Mendelian randomization (MR) uses genetic variants associated with the factor of interest (e.g. cognition) to test the hypothesis that this factor increases the risk of developing a particular outcome (e.g. obesity) [71]. Using this method, one study found a causal effect of waist to hip ratio adjusted for BMI to impair cognition but did not assess the reverse effect of cognition on adiposity [72]. Two studies found a negative causal effect of educational attainment (which may be a proxy for cognitive ability) on BMI [73,74], whereas another found no causal association between educational ability and BMI, although the authors noted that the study was underpowered [75]. Another genetically informed approach is direction of causation (DoC) twin modelling, which uses data observed in monozygotic and dizygotic twins to test causal hypotheses. The approach is based on the premise that different cross-twin/cross-trait covariances are expected for different types of causal models [76]. Future studies should examine causal effects of specific cognitive abilities on BMI and other measures of adiposity using the MR and DoC approaches.

Experimental designs involving randomization and manipulation of a hypothesized causal factor provide the strongest basis for making causal inference. Interventions that have been used to assess causal relationships between higher cognitive functions and obesity include cognitive training, weight loss interventions and manipulations of cognitive performance and diet in non-human animal models.

## 7. Effects of cognitive training on food intake and body weight

Cognitive training interventions involve repeated completion of digital based tasks that engage a specific cognitive function over several weeks [77]. Training of executive functions can lead to significant improvements in performance of the trained tasks with evidence for transfer to non-trained tasks [78]. The causal effect of cognitive function on obesity can, therefore, be tested by examining whether enhancing cognitive function via practice prevents weight gain or assists with weight management. Studies conducted to date have focused on first establishing whether cognitive training affects food intake as an intervening variable in the potential causal chain between cognition and weight gain and development of obesity. The findings from two reviews suggest that cognitive training of working memory, episodic future thinking and food-specific inhibitory control, results in a decrease short-term food intake [79,80]. However, at present there is not sufficient evidence to suggest that these effects are translated into a reduction body weight either in adults or children [81,82].

The findings on cognitive training need to be interpreted in the light of limitations to the studies conducted to date. Most studies have been conducted on small populations of normal weight adult participants and so the lack of effects of training on body weight may be explained by several factors, including whether the training is appropriately targeted at individuals who might benefit the most, e.g. participants with obesity and lower baseline levels of performance [79]. Future studies should also ensure that the training is engaging enough to maintain adherence [18,19]. It is possible that training may be more effective if it occurs during neurodevelopmental periods when the brain is maturing. During adolescence, the brain undergoes extensive remodelling especially in regions associated with higher cognitive functions such as the prefrontal cortex, which means it is sensitive to environmental influences at this point. Hence, future interventions could target adolescent populations at high risk of obesity to examine whether training could prevent weight gain [83], especially as young people may be more vulnerable to effects of diet and obesity on the brain [84].

## 8. Effects of weight loss on cognition

The causal pathway from obesity to cognitive function can be tested by examining the effect of weight loss interventions on cognitive function. Weight loss is associated with improved verbal memory and executive function in adults [85]. A meta-analysis of both observational longitudinal and randomized controlled trials of a range of weight loss interventions, including bariatric surgery, found that weight loss was associated with improved attention and memory (working memory and hippocampal-dependent memory), at least in the short term [86]. However, there is large variability across studies and the underlying mechanisms are unclear. Weight loss is associated with an improvement in metabolic indicators related to cognitive function but there are also changes in emotional functioning and mental health outcomes that accompany weight loss that could also explain improvements in cognition [87].

Studies of changes in brain structure following bariatric surgery suggest that the improvements seen in cognitive function may be owing to recovery of obesity-associated brain atrophy. Increases in both grey and WM density have been observed post-surgery [88–91]. Widespread recovery of WM and smaller increases in GM have been noted, which could indicate that longer-term assessment is required to see GM recovery, but could also indicate that some obesity-associated reductions in GM reflect differences in brain structure that predispose towards obesity and are not recoverable by weight loss. These data suggest that having obesity affects brain structure integrity, which probably underlies the cognitive profile seen in people with obesity, and that at least some of these effects are reversible.

## 9. Non-human animal models

Intervention studies in laboratory animals allow for tight control over parameters such as diet history, food access and housing conditions as well as precise measurement of food intake and body weight over lengthy periods, which is not easy to achieve when studying humans. It has been established that disruption to memory processes, via lesions or temporary inactivation of the hippocampus, results in an increase in food intake and subsequent weight gain [92–95]. Evidence from animal models also confirms the involvement of brain structures important for higher cognitive functions in the control of food intake (for review see [96]). Furthermore, evidence from animal models shows that diet influences behaviour and brain structure and function [97,98]. Exposure to high energy diet and/or obesity impairs hippocampal-related memory performance in rodents [99,100] owing to changes to neuronal signalling in the hippocampus [101] and areas of the brain homologous to the prefrontal cortex in humans [102]. Hence, the data from animal models supports a causal effect of cognition, particularly hippocampal-related memory on the development of obesity as well as the reverse effect [103,104]. It is notable that the effects of diet on cognitive processes in non-human animal models are much more profound than the more subtle cognitive impairments associated with obesity in humans. Such differences in the magnitude of the effects in non-human relative to human animal models may reflect differences in the sensitivity of the measures of the processes, differences in the types of cognitive processes that are being assessed in the two species, and/or the magnitude of the exposure.

## 10. Gaps in knowledge and future directions

At present, the relative influence of genetic compared to environmental factors as causes of the association between higher cognitive functions and obesity is unclear. Genetic correlations between cognitive test scores and brain morphometry and BMI have been reported cross-sectionally [105], which could suggest that inherited variability in cognitive function underlies the association with obesity. However, a longitudinal study found a significant impact of overweight, but not genetic predisposition for obesity on altered brain structure [106]. The genetic analysis by Tüngler and colleagues was underpowered and so fully powered longitudinal studies are required to test the hypothesis that genetic risk for obesity is expressed through alterations in cognitive function.

Environmental factors such as adversity and stress cause disruption to cognitive processes which could then lead to the development of obesity. Experiencing childhood poverty and a range of other early adverse childhood experiences (e.g. exposure to harsh, unpredictable environments) are related to poorer cognitive function, altered trajectories of brain development and poorer physical and mental health outcomes [107–109]. Notably, moving into poverty has been linked to worsening executive function in childhood and lower teacher rated self-regulatory ability, whereas moving out of poverty was associated with the opposite effects [110]. In addition, exposure to stressors (including structural racism and weight stigma) and depression are linked to poorer cognitive function and health outcomes [35,36]. Hence, further work is required to examine the mediating role that cognitive function might play in the effect that living in poverty, trauma and mental health conditions have on obesity.

It is important to note that the differences in cognitive function according to body weight status are small, meaning that many people with obesity do not differ in cognitive function from people without obesity [37]. It is possible that a subset of individuals who develop obesity do so because of having a particular cognitive phenotype that promotes food intake. It is also possible that the effect sizes detected in studies reported to date are an underestimation of the true effect size owing to the nature of the cognitive measures employed. Most studies use measures that tap into general cognitive ability that are far removed from how these cognitive functions are deployed in real life decisions about eating. Such decisions take place in the context of prior experience with specific foods and in a context that is rich in information, including knowledge about the nutritional content of food, branding, and social cues. Moreover, decisions about whether, what, when and how much to eat require the interaction/integration of multiple cognitive processes. It is possible that assessment of cognition in situations that are more representative of how cognitive processes are usually situated would uncover not only larger effect sizes but shed light on the specific underlying cognitive processes involved in food choices that predispose towards obesity.

The availability of large datasets for testing longitudinal associations between cognition and obesity, especially in children, is a step forward from cross-sectional research but these datasets often lack measures related to eating (or physical activity). The inclusion of such measures in future studies is required to link cognition mechanistically to obesity through changes in health behaviours. As reduced higher cognitive function has been found to predict both unhealthier eating patterns and lower levels of physical activity [17,111], separating out the relative contribution of both sides of the energy balance equation to weight gain over time would be a useful avenue for future research. In addition, it would be informative to examine associations between higher cognitive functions and specific eating patterns. A recent meta-analysis found evidence for an association between executive functioning (studies mainly focusing on inhibitory control) and clinical binge eating, but not uncontrolled eating [112]. However, the analysis of uncontrolled eating was underpowered owing to the low number of studies included. Therefore, there is scope for assessing relationships between higher cognitive processes and patterns of eating, including uncontrolled eating, but also emotional eating and eating triggered by external cues.

It is also important to note the paucity of studies on higher cognitive functions outside of the classical executive functions that are underpinned by prefrontal cortex activity. Further research on the contribution of hippocampal-dependent processes is warranted [113].

Intervention studies would provide more definitive conclusions about causal association between cognition and obesity in humans. For example, studies conducted in a controlled laboratory environment could test whether cognitive disruption to food-related decision making increases intake in the longer term. Many studies have found that distractions, e.g. watching TV or playing computer games are associated with increased intake both in a meal with distractions and at later snacking opportunities [114,115]. Such effects may be explained by reduced cognitive capacity for decision making as well as poorer meal memory encoding. However, these studies have only assessed the effects of distraction on short-term intake and so it is unknown whether there is compensation in the longer term. Sustained effects of distraction on intake would support a role for higher cognition in the development of obesity in line with observational evidence that daily distracted consumption patterns are positively associated with obesity [116].

Education improves cognitive performance in specific domains such as memory and reasoning [117] and so education interventions can be used to manipulate cognitive function and might prove to be a tool to reduce obesity risk. Indeed, taking advantage of a natural experiment that occurred in the UK in 1972 when the school leaving age was raised by one year, Barcellos *et al.* [118] showed that this policy change was associated with a reduction in levels of obesity, with a larger effect for individuals with high genetic predisposition to obesity. The positive effect of education on obesity is also supported by a meta-analysis of studies of changes to compulsory schooling laws [119]. These data suggest that interventions which increase/improve educational attainment could reduce levels of obesity and that one way in which this might occur is via improvement in cognition. The effects of education interventions (e.g. meta-cognition and self-regulation strategies/social–emotional education) on both attainment, cognition and health outcomes could be tested.

Cognitive function can be manipulated using pharmacological interventions. Lisdexamphetamine (LDX) has been marketed for several years for the treatment of cognitive symptoms of attention deficit hyperactivity disorder (ADHD) and more recently in some countries as treatment for binge eating disorder. LDX has been reported to improve inhibitory control in women with binge eating symptoms and, alongside this improvement in cognition, to reduce food intake [120]. The effects of LDX and other cognitive enhancers on weight loss/prevention of weight gain could be examined in randomized controlled studies [121,122]. Interestingly, ADHD is associated with disinhibited eating and obesity [123,124] and this relationship is related to deficits in attention and cognitive control [124,125]. Moreover, rates of obesity are lower in individuals who are pharmacologically treated for ADHD compared with those who are not treated [123]. Further investigation of the development of obesity in ADHD and other psychiatric disorders associated with cognitive problems and obesity will shed further light on the role of specific cognitive deficits in weight gain.

## 11. Conclusion

There is a plausible, theoretically grounded, mechanistic link between cognition and obesity: disruption to higher cognitive functions increases food intake which could result positive energy balance in the longer term. Most research aimed at testing whether disruption to higher cognitive processes causes obesity has involved investigation of cross-sectional associations between higher cognitive functions and obesity. There is now ample evidence that high levels of adiposity are associated with lower cognitive performance on tasks assessing working memory and inhibitory control. Further evidence on this point is unlikely to be informative. Data from longitudinal and interventional studies and from non-human animal models suggest that a reciprocal relationship between obesity and cognitive function exists but have not yet established cognition as a primary cause of obesity in humans. Longitudinal studies that start from an early age and include eating-related measures as well as a range of cognitive tests and measures of adiposity will provide new insights into the specific cognitive factors that might predispose some people to develop obesity and/or exacerbate weight gain. Other approaches, including genetically informed studies and randomized interventions aimed manipulating cognitive function could also prove fruitful.

## Data Availability

This article has no additional data.

## References

[1] Di Angelantonio E et al. 2016 Body-mass index and all-cause mortality: individual-participant-data meta-analysis of 239 prospective studies in four continents. Lancet **388**, 776-786. (10.1016/S0140-6736(16)30175-1) 27423262 PMC4995441

[2] Higgs S, Spetter MS. 2018 Cognitive control of eating: the role of memory in appetite and weight gain. Curr. Obes. Rep. **7**, 50-59. (10.1007/s13679-018-0296-9) 29430616 PMC5829122

[3] Lowe CJ, Staines WR, Manocchio F, Hall PA. 2018 The neurocognitive mechanisms underlying food cravings and snack food consumption. A combined continuous theta burst stimulation (cTBS) and EEG study. Neuroimage **177**, 45-58. (10.1016/j.neuroimage.2018.05.013) 29742385

[4] Zhang Z, Coppin G. 2018 To what extent memory could contribute to impaired food valuation and choices in obesity? Front. Psychol. **9**, 2523. (10.3389/fpsyg.2018.02523) 30618948 PMC6297373

[5] Diamond A. 2013 Executive functions. Annu. Rev. Psychol. **64**, 135. (10.1146/annurev-psych-113011-143750) 23020641 PMC4084861

[6] Lengyel M, Dayan P. 2007 Hippocampal contributions to control: the third way. *In* Proc. 20th Int. Conf. on Neural Information Processing Systems, December 2007, pp. 889-896. Red Hook, NY: Curran Associates Inc.

[7] Higgs S. 2016 Cognitive processing of food rewards. Appetite **104**, 10-17. (10.1016/j.appet.2015.10.003) 26458961

[8] Daly M, McMinn D, Allan JL. 2015 A bidirectional relationship between physical activity and executive function in older adults. Front. Hum. Neurosci. **8**, 1044. (10.3389/fnhum.2014.01044) 25628552 PMC4292779

[9] Hall KD. 2018 Did the food environment cause the obesity epidemic? Obesity **26**, 11-13. (10.1002/oby.22073) 29265772 PMC5769871

[10] Hofmann W, Friese M, Strack F. 2009 Impulse and self-control from a dual-systems perspective. Perspect. Psychol. Sci. **4**, 162-176. (10.1111/j.1745-6924.2009.01116.x) 26158943

[11] Hare TA et al. 2009 Self-control in decision-making involves modulation of the vmPFC valuation system. Science **324**, 646-648. (10.1126/science.1168450) 19407204

[12] Han JE et al. 2018 Neural correlates of dietary self-control in healthy adults: a meta-analysis of functional brain imaging studies. Physiol. Behav. **192**, 98-108. (10.1016/j.physbeh.2018.02.037) 29496487

[13] Berkman ET, Hutcherson CA, Livingston JL, Kahn LE, Inzlicht M. 2017 Self-control as value-based choice. Curr. Dir. Psychol. Sci. **26**, 422-428. (10.1177/0963721417704394) 29335665 PMC5765996

[14] Higgs S. 2005 Memory and its role in appetite regulation. Physiol. Behav. **85**, 67-72. (10.1016/j.physbeh.2005.04.003) 15924907

[15] Rustichini A. 2021 Working memory and attention in choice. See https://ssrn.com/abstract=3964802.

[16] Allom V, Mullan B. 2014 Individual differences in executive function predict distinct eating behaviours. Appetite **80**, 123-130. (10.1016/j.appet.2014.05.007) 24845785

[17] Riggs NR, Spruijt-Metz D, Sakuma KL, Chou CP, Pentz MA. 2010 Executive cognitive function and food intake in children. J. Nutr. Educ. Behav. **42**, 398-403. (10.1016/j.jneb.2009.11.003) 20719568 PMC2980564

[18] Whitelock V, Nouwen A, van den Akker O, Higgs S. 2018 The role of working memory sub-components in food choice and dieting success. Appetite **124**, 24-32. (10.1016/j.appet.2017.05.043) 28554850

[19] Whitelock V, Nouwen A, Houben K, van den Akker O, Rosenthal M, Higgs S. 2018 Does working memory training improve dietary self-care in type 2 diabetes mellitus? Results of a double blind randomised controlled trial. Diabetes Res. Clin. Pract. **143**, 204-214. (10.1016/j.diabres.2018.07.005) 30017630

[20] Higgs S, Williamson AC, Rotshtein P, Humphreys GW. 2008 Sensory-specific satiety is intact in amnesics who eat multiple meals. Psychol. Sci. **19**, 623-628. (10.1111/j.1467-9280.2008.02132.x) 18727773

[21] Parent MB, Higgs S, Cheke LG, Kanoski SE. 2022 Memory and eating: a bidirectional relationship implicated in obesity. Neurosci. Biobehav. Rev. **132**, 110-129. (10.1016/j.neubiorev.2021.10.051) 34813827 PMC8816841

[22] Amlung M, Petker T, Jackson J, Balodis I, MacKillop J. 2016 Steep discounting of delayed monetary and food rewards in obesity: a meta-analysis. Psychol. Med. **46**, 2423-2434. (10.1017/S0033291716000866) 27299672

[23] Lavagnino L, Arnone D, Cao B, Soares JC, Selvaraj S. 2016 Inhibitory control in obesity and binge eating disorder: a systematic review and meta-analysis of neurocognitive and neuroimaging studies. Neurosci. Biobehav. Rev. **68**, 714-726. (10.1016/j.neubiorev.2016.06.041) 27381956

[24] Yang Y, Shields GS, Guo C, Liu Y. 2018 Executive function performance in obesity and overweight individuals: a meta-analysis and review. Neurosci. Biobehav. Rev. **84**, 225-244. (10.1016/j.neubiorev.2017.11.020) 29203421

[25] Wu M, Brockmeyer T, Hartmann M, Skunde M, Herzog W, Friederich HC. 2016 Reward-related decision making in eating and weight disorders: a systematic review and meta-analysis of the evidence from neuropsychological studies. Neurosci. Biobehav. Rev. **61**, 177-196. (10.1016/j.neubiorev.2015.11.017) 26698021

[26] Liang J, Matheson BE, Kaye WH, Boutelle KN. 2014 Neurocognitive correlates of obesity and obesity-related behaviors in children and adolescents. Int. J. Obes. **38**, 494-506. (10.1038/ijo.2013.142) PMC4456183 23913029

[27] Davidson TL et al. 2022 Retrieval-induced forgetting in children and adolescents with and without obesity. Int. J. Obes. **46**, 851-858. (10.1038/s41366-021-01036-5) PMC8967761 35042933

[28] Mamrot P, Hanć T. 2019 The association of the executive functions with overweight and obesity indicators in children and adolescents: a literature review. Neurosci. Biobehav. Rev. **107**, 59-68. (10.1016/j.neubiorev.2019.08.021) 31470031

[29] Loprinzi PD, Frith E. 2018 Obesity and episodic memory function. J. Physiol. Sci. **68**, 321-331. (10.1007/s12576-018-0612-x) 29667132 PMC10717800

[30] Cheke LG, Simons JS, Clayton NS. 2016 Higher body mass index is associated with episodic memory deficits in young adults. Q. J. Exp. Psychol. (Colchester) **69**, 2305-2316. (10.1080/17470218.2015.1099163) PMC5000869 26447832

[31] Coppin G, Nolan-Poupart S, Jones-Gotman M, Small DM. 2014 Working memory and reward association learning impairments in obesity. Neuropsychologia **65**, 146-155. (10.1016/j.neuropsychologia.2014.10.004) 25447070 PMC4259845

[32] Lynch KM, Page KA, Shi Y, Xiang AH, Toga AW, Clark KA. 2021 The effect of body mass index on hippocampal morphology and memory performance in late childhood and adolescence. Hippocampus **31**, 189-200. (10.1002/hipo.23280) 33174346 PMC9006989

[33] Anand SS et al. 2022 Evaluation of adiposity and cognitive function in adults. JAMA Netw. Open **5**, e2146324. (10.1001/jamanetworkopen.2021.46324) 35103790 PMC8808326

[34] Hartanto A, Yong JC. 2018 Measurement matters: higher waist-to-hip ratio but not body mass index is associated with deficits in executive functions and episodic memory. PeerJ **6**, e5624. (10.7717/peerj.5624) 30210946 PMC6130234

[35] Webb EK, Cardenas-Iniguez C, Douglas R. 2022 Radically reframing studies on neurobiology and socioeconomic circumstances: a call for social justice-oriented neuroscience. Front. Integr. Neurosci. **16**, 958545. (10.3389/fnint.2022.958545) 36118113 PMC9479322

[36] Chen R, Weuve J, Misra S, Cuevas A, Kubzansky LD, Williams DR. 2022 Racial disparities in cognitive function among middle-aged and older adults: the roles of cumulative stress exposures across the life course. J. Gerontol. A Biol. Sci. Med. Sci. **77**, 357-364. (10.1093/gerona/glab099) 33824971 PMC8824673

[37] Robinson E, Roberts C, Vainik U, Jones A. 2020 The psychology of obesity: an umbrella review and evidence-based map of the psychological correlates of heavier body weight. Neurosci. Biobehav. Rev. **119**, 468-480. (10.1016/j.neubiorev.2020.10.009) 33086131

[38] Parsons N, Steward T, Clohesy R, Almgren H, Duehlmeyer L. 2021 A systematic review of resting-state functional connectivity in obesity: refining current neurobiological frameworks and methodological considerations moving forward. Rev. Endocr. Metab. Disord. **23**, 861-879. (10.1007/s11154-021-09665-x) 34159504

[39] Syan SK, McIntyre-Wood C, Minuzzi L, Hall G, McCabe RE, MacKillop J. 2021 Dysregulated resting state functional connectivity and obesity: a systematic review. Neurosci. Biobehav. Rev. **131**, 270-292. (10.1016/j.neubiorev.2021.08.019) 34425125

[40] Park BY et al. 2021 Inter-individual body mass variations relate to fractionated functional brain hierarchies. Commun. Biol. **4**, 1-12. (10.1038/s42003-020-01566-0) 34127795 PMC8203627

[41] Brooks SJ, Cedernaes J, Schiöth HB. 2013 Increased prefrontal and parahippocampal activation with reduced dorsolateral prefrontal and insular cortex activation to food images in obesity: a meta-analysis of fMRI studies. PLoS One **8**, e60393. (10.1371/journal.pone.0060393) 23593210 PMC3622693

[42] Devoto F, Zapparoli L, Bonandrini R, Berlingeri M, Ferrulli A, Luzi L, Banfi G, Paulesu E. 2018 Hungry brains: a meta-analytical review of brain activation imaging studies on food perception and appetite in obese individuals. Neurosci. Biobehav. Rev. **94**, 271-285. (10.1016/j.neubiorev.2018.07.017) 30071209

[43] Chen EY, Eickhoff SB, Giovannetti T, Smith DV. 2020 Obesity is associated with reduced orbitofrontal cortex volume: a coordinate-based meta-analysis. NeuroImage Clin. **28**, 102420. (10.1016/j.nicl.2020.102420) 32961404 PMC7509458

[44] García-García I, Michaud A, Dadar M, Zeighami Y, Neseliler S, Collins DL, Evans AC, Dagher A. 2019 Neuroanatomical differences in obesity: meta-analytic findings and their validation in an independent dataset. Int. J. Obes. **43**, 943-951. (10.1038/s41366-018-0164-4) 30022057

[45] Herrmann MJ, Tesar AK, Beier J, Berg M, Warrings B. 2019 Grey matter alterations in obesity: a meta-analysis of whole-brain studies. Obes. Rev. **20**, 464-471. (10.1111/obr.12799) 30537231

[46] Zapparoli L, Devoto F, Giannini G, Zonca S, Gallo F, Paulesu E. 2022 Neural structural abnormalities behind altered brain activation in obesity: evidence from meta-analyses of brain activation and morphometric data. NeuroImage Clin. **36**, 103179. (10.1016/j.nicl.2022.103179) 36088842 PMC9474923

[47] Laurent JS, Watts R, Adise S, Allgaier N, Chaarani B, Garavan H, Potter A, Mackey S. 2020 Associations among body mass index, cortical thickness, and executive function in children. J. Am. Med. Assoc. Pediatr. **174**, 170-177. (10.1001/jamapediatrics.2019.4708) PMC6902097 31816020

[48] Ronan L, Alexander-Bloch A, Fletcher PC. 2020 Childhood obesity, cortical structure, and executive function in healthy children. Cereb. Cortex **30**, 2519-2528. (10.1093/cercor/bhz257) 31646343 PMC7175011

[49] Morys F, Shishikura M, Dagher A. 2022 Population-based research in obesity–an overview of neuroimaging studies using big data approach. Curr. Opin. Endocr. Metab. Res. **23**, 100323. (10.1016/j.coemr.2022.100323)

[50] Kullmann S, Schweizer F, Veit R, Fritsche A, Preissl H. 2015 Compromised white matter integrity in obesity. Obes. Rev. **16**, 273-281. (10.1111/obr.12248) 25676886

[51] Daoust J, Schaffer J, Zeighami Y, Dagher A, García-García I, Michaud A. 2021 White matter integrity differences in obesity: a meta-analysis of diffusion tensor imaging studies. Neurosci. Biobehav. Rev. **129**, 133-141. (10.1016/j.neubiorev.2021.07.020) 34284063

[52] Okudzhava L, Heldmann M, Münte TF. 2022 A systematic review of diffusion tensor imaging studies in obesity. Obes. Rev. **23**, e13388. (10.1111/obr.13388) 34908217

[53] Pflanz CP, Tozer DJ, Harshfield EL, Tay J, Farooqi S, Markus HS. 2022 Central obesity is selectively associated with cerebral gray matter atrophy in 15,634 subjects in the UK Biobank. Int. J. Obes. **46**, 1059-1067. (10.1038/s41366-021-00992-2) PMC9050590 35145215

[54] Nouwen A, Chambers A, Chechlacz M, Higgs S, Blissett J, Barrett TG, Allen HA. 2017 Microstructural abnormalities in white and gray matter in obese adolescents with and without type 2 diabetes. NeuroImage Clin. **16**, 43-51. (10.1016/j.nicl.2017.07.004) 28752059 PMC5514690

[55] Whitelock V, Rutters F, Rijnhart JJ, Nouwen A, Higgs S. 2021 The mediating role of comorbid conditions in the association between type 2 diabetes and cognition: a cross-sectional observational study using the UK Biobank cohort. Psychoneuroendocrinology **123**, 104902. (10.1016/j.psyneuen.2020.104902) 33197721

[56] Forte N et al. 2021 Orexin-A and endocannabinoids are involved in obesity-associated alteration of hippocampal neurogenesis, plasticity, and episodic memory in mice. Nat. Commun. **12**, 1-20. (10.1038/s41467-021-26388-4) 34675233 PMC8531398

[57] Sharma S. 2021 High fat diet and its effects on cognitive health: alterations of neuronal and vascular components of brain. Physiol. Behav. **240**, 113528. (10.1016/j.physbeh.2021.113528) 34260890

[58] Taylor ZB, Stevenson RJ, Ehrenfeld L, Francis HM. 2021 The impact of saturated fat, added sugar and their combination on human hippocampal integrity and function: a systematic review and meta-analysis. Neurosci. Biobehav. Rev. **130**, 91-106. (10.1016/j.neubiorev.2021.08.008) 34400179

[59] Yang Y, Shields GS, Wu Q, Liu Y, Chen H, Guo C. 2020 The association between obesity and lower working memory is mediated by inflammation: findings from a nationally representative dataset of US adults. Brain Behav. Immun. **84**, 173-179. (10.1016/j.bbi.2019.11.022) 31785398

[60] Anzman SL, Birch LL. 2009 Low inhibitory control and restrictive feeding practices predict weight outcomes. J. Pediatr. **155**, 651-656. (10.1016/j.jpeds.2009.04.052) 19595373 PMC2764786

[61] Francis LA, Susman EJ. 2009 Self-regulation and rapid weight gain in children from age 3 to 12 years. Arch. Pediatr. Adolesc. Med. **163**, 297-302. (10.1001/archpediatrics.2008.579) 19349557 PMC9433163

[62] Cappelli C, Pike JR, Riggs NR, Warren CM, Pentz MA. 2019 Executive function and probabilities of engaging in long-term sedentary and high calorie/low nutrition eating behaviors in early adolescence. Soc. Sci. Med. **237**, 112483. (10.1016/j.socscimed.2019.112483) 31404882 PMC6711174

[63] Jernigan TL, Brown SA, Dowling GJ. 2018 The adolescent brain cognitive development study. J. Res. Adolesc. **28**, 154. 29460352 10.1111/jora.12374 PMC7477916

[64] Hall PA, Best JR, Beaton EA, Sakib MN, Danckert J. 2021 Morphology of the prefrontal cortex predicts body composition in early adolescence: cognitive mediators and environmental moderators in the ABCD Study. Soc. Cogn. Affect. Neurosci. **18**, nsab104. (10.1093/scan/nsab104) PMC11305164 34471927

[65] Jiang F et al. 2022 Obesity is associated with decreased gray matter volume in children: a longitudinal study. Cereb. Cortex **33**, 3674-3682. (10.1093/cercor/bhac300) PMC10068275 35989308

[66] Adise S, Marshall AT, Hahn S, Zhao S, Kan E, Rhee K, Herting MM, Sowell ER. 2022 Longitudinal assessment of brain structure and behavior in youth with rapid weight gain: potential contributing causes and consequences. Paediatr. Obes. **18**, e12985. 10.1111/ijpo.12985 PMC11075780 36253967

[67] Adise S et al. 2021 Multimodal brain predictors of current weight and weight gain in children enrolled in the ABCD study®. Dev. Cogn. Neurosci. **49**, 100948. (10.1016/j.dcn.2021.100948) 33862325 PMC8066422

[68] Shields GS, Deer LK, Hastings PD, Hostinar CE. 2021 Adiposity, inflammation, and working memory: evidence for a vicious cycle. Brain Behav. Immun.-Health **13**, 100202. (10.1016/j.bbih.2021.100202) 33899030 PMC8061900

[69] Likhitweerawong N, Louthrenoo O, Boonchooduang N, Tangwijitsakul H, Srisurapanont M. 2022 Bidirectional prediction between weight status and executive function in children and adolescents: a systematic review and meta-analysis of longitudinal studies. Obes. Rev. **23**, e13458. (10.1111/obr.13458) 35508917

[70] Nazmus Sakib M, Best JR, Ramezan R, Thompson ME, & Hall PA. 2022 Bidirectional associations between adiposity and cognitive function: a prospective analysis of the Canadian Longitudinal Study on Aging (CLSA). J. Gerontol. A Biol. Sci. Med. Sci. **78**, 314-325. (10.1093/gerona/glac115) PMC9951058 35640256

[71] Davey Smith G, & Ebrahim S. 2003 ‘Mendelian randomization’: can genetic epidemiology contribute to understanding environmental determinants of disease? Int. J. Epidemiol. **32**, 1-22. (10.1093/ije/dyg070) 12689998

[72] Wang SH, Su MH, Chen CY, Lin YF, Feng YC, Hsiao PC, Pan YJ, Wu CS. 2022 Causality of abdominal obesity on cognition: a trans-ethnic Mendelian randomization study. Int. J. Obes. **46**, 1487-1492. (10.1038/s41366-022-01138-8) 35538205

[73] Böckerman P, Viinikainen J, Pulkki-Råback L, Hakulinen C, Pitkänen N, Lehtimäki T, Pehkonen J, Raitakari OT. 2017 Does higher education protect against obesity? Evidence using Mendelian randomization. Prev. Med. **101**, 195-198. (10.1016/j.ypmed.2017.06.015) 28645627

[74] Cao M, Cui B. 2020 Association of educational attainment with adiposity, type 2 diabetes, and coronary artery diseases: a Mendelian randomization study. Front. Public Health **8**, 112. (10.3389/fpubh.2020.00112) 32391302 PMC7189805

[75] Hagenaars SP et al. 2016 Shared genetic aetiology between cognitive functions and physical and mental health in UK Biobank (N= 112 151) and 24 GWAS consortia. Mol. Psychiatry **21**, 1624-1632. (10.1038/mp.2015.225) 26809841 PMC5078856

[76] Briley DA, Livengood J, Derringer J. 2018 Behaviour genetic frameworks of causal reasoning for personality psychology. Eur. J. Pers. **32**, 202-220. (10.1002/per.2153)

[77] von Bastian CC, Belleville S, Udale RC, Reinhartz A, Essounni M, Strobach T. 2022 Mechanisms underlying training-induced cognitive change. Nat. Rev. Psychol. **1**, 30-41. (10.1038/s44159-021-00001-3) PMC9270821 35804410

[78] Karbach J, Verhaeghen P. 2014 Making working memory work: a meta-analysis of executive-control and working memory training in older adults. Psychol. Sci. **25**, 2027-2037. (10.1177/0956797614548725) 25298292 PMC4381540

[79] Jones A, Hardman CA, Lawrence N, Field M. 2018 Cognitive training as a potential treatment for overweight and obesity: a critical review of the evidence. Appetite **124**, 50-67. (10.1016/j.appet.2017.05.032) 28546010

[80] Yang Y, Shields GS, Wu Q, Liu Y, Chen H, Guo C. 2019 Cognitive training on eating behaviour and weight loss: a meta-analysis and systematic review. Obes. Rev. **20**, 1628-1641. (10.1111/obr.12916) 31353774

[81] Luis-Ruiz S, Caldú X, Sánchez-Castañeda C, Pueyo R, Garolera M, Jurado MÁ. 2020 Is cognitive training an effective tool for improving cognitive function and real-life behaviour in healthy children and adolescents? A systematic review. Neurosci. Biobehav. Rev. **116**, 268-282. (10.1016/j.neubiorev.2020.06.019) 32565174

[82] Szabo-Reed AN, Donnelly JE. 2021 Cognitive training: associations and implications for weight management and translational research. Transl. J. Am. Coll. Sports Med. **6**, e000151. 34017915 10.1249/tjx.0000000000000151 PMC8130573

[83] Eichen DM, Appleton-Knapp S, Boutelle KN. 2018 Childhood obesity and cognitive function. In Pediatric obesity: ethology, pathogenesis and treatment (ed. MS Freemark), pp. 539-554. New York, NY: Springer.

[84] Lowe CJ, Morton JB, Reichelt AC. 2020 Adolescent obesity and dietary decision making—a brain-health perspective. Lancet Child Adolesc. Health **4**, 388-396. (10.1016/S2352-4642(19)30404-3) 32164832

[85] Veronese N et al. 2017 Weight loss is associated with improvements in cognitive function among overweight and obese people: a systematic review and meta-analysis. Neurosci. Biobehav. Rev. **72**, 87-94. (10.1016/j.neubiorev.2016.11.017) 27890688

[86] Siervo M, Arnold R, Wells JC, Tagliabue A, Colantuoni A, Albanese E, Brayne C, Stephan BC. 2011 Intentional weight loss in overweight and obese individuals and cognitive function: a systematic review and meta-analysis. Obes. Rev. **12**, 968-983. (10.1111/j.1467-789X.2011.00903.x) 21762426

[87] Patsalos O, Keeler J, Schmidt U, Penninx BW, Young AH, Himmerich H. 2021 Diet, obesity, and depression: a systematic review. J. Pers. Med. **11**, 176. (10.3390/jpm11030176) 33802480 PMC7999659

[88] Michaud A et al. 2020 Neuroanatomical changes in white and grey matter after sleeve gastrectomy. Neuroimage **213**, 116696. (10.1016/j.neuroimage.2020.116696) 32145436

[89] Tuulari JJ et al. 2016 Bariatric surgery induces white and grey matter density recovery in the morbidly obese: a voxel-based morphometric study. Hum. Brain Mapp. **37**, 3745-3756. (10.1002/hbm.23272) 27400738 PMC6867305

[90] Zhang Y et al. 2016 Recovery of brain structural abnormalities in morbidly obese patients after bariatric surgery. Int. J. Obes. **40**, 1558-1565. (10.1038/ijo.2016.98) 27200505

[91] Zeighami Y et al. 2022 Impact of weight loss on brain age: improved brain health following bariatric surgery. Neuroimage **259**, 119415. (10.1016/j.neuroimage.2022.119415) 35760293

[92] Clifton PG, Vickers SP, Somerville EM. 1998 Little and often: ingestive behavior patterns following hippocampal lesions in rats. Behav. Neurosci. **112**, 502. (10.1037/0735-7044.112.3.502) 9676968

[93] Davidson TL, Kanoski SE, Walls EK, Jarrard LE. 2005 Memory inhibition and energy regulation. Physiol. Behav. **86**, 731-746. (10.1016/j.physbeh.2005.09.004) 16263144

[94] Henderson YO, Smith GP, Parent MB. 2013 Hippocampal neurons inhibit meal onset. Hippocampus **23**, 100-107. (10.1002/hipo.22062) 22927320

[95] Hannapel R, Ramesh J, Ross A, LaLumiere RT, Roseberry AG, Parent MB. 2019 Postmeal optogenetic inhibition of dorsal or ventral hippocampal pyramidal neurons increases future intake. Eneuro **6**, ENEURO.0457-18.2018. (10.1523/ENEURO.0457-18.2018) PMC6348449 30693314

[96] Azevedo EP, Ivan VJ, Friedman JM, Stern SA. 2021 Higher-order inputs involved in appetite control. Biol. Psychiatry **91**, 869-878. 34593204 10.1016/j.biopsych.2021.07.015 PMC9704062

[97] Kanoski SE, Davidson TL. 2011 Western diet consumption and cognitive impairment: links to hippocampal dysfunction and obesity. Physiol. Behav. **103**, 59-68. (10.1016/j.physbeh.2010.12.003) 21167850 PMC3056912

[98] Reichelt AC, Stoeckel LE, Reagan LP, Winstanley CA, Page KA. 2018 Dietary influences on cognition. Physiol. Behav. **192**, 118-126. (10.1016/j.physbeh.2018.02.052) 29501837 PMC6071323

[99] Greenwood CE, Winocur G. 1990 Learning and memory impairment in rats fed a high saturated fat diet. Behav. Neur. Biol. **53**, 74-87. (10.1016/0163-1047(90)90831-P) 2302144

[100] Kanoski SE, Davidson TL. 2010 Different patterns of memory impairments accompany short-and longer-term maintenance on a high-energy diet. J. Exp. Psychol. Anim. Behav. Process. **36**, 313. (10.1037/a0017228) 20384410

[101] Stranahan AM, Norman ED, Lee K, Cutler RG, Telljohann RS, Egan JM, Mattson MP. 2008 Diet-induced insulin resistance impairs hippocampal synaptic plasticity and cognition in middle-aged rats. Hippocampus **18**, 1085-1088. (10.1002/hipo.20470) 18651634 PMC2694409

[102] Baker KD, Reichelt AC. 2016 Impaired fear extinction retention and increased anxiety-like behaviours induced by limited daily access to a high-fat/high-sugar diet in male rats: implications for diet-induced prefrontal cortex dysregulation. Neurobiol. Learn. Mem. **136**, 127-138. (10.1016/j.nlm.2016.10.002) 27720810

[103] Hargrave SL, Jones S, Davidson TL. 2016 The outward spiral: a vicious cycle model of obesity and cognitive dysfunction. Curr. Opin. Behav. Sci. **9**, 40-46. (10.1016/j.cobeha.2015.12.001) 26998507 PMC4795925

[104] Davidson TL, Stevenson RJ. 2022 Appetitive interoception, the hippocampus and western-style diet. Rev. Endocr. Metab. Disord. **23**, 845-859. (10.1007/s11154-021-09698-2) 35067848

[105] Vainik U et al. 2018 Neurobehavioral correlates of obesity are largely heritable. Proc. Natl Acad. Sci. USA **115**, 9312-9317. (10.1073/pnas.1718206115) 30154161 PMC6140494

[106] Tüngler A et al. 2021 Body mass index but not genetic risk is longitudinally associated with altered structural brain parameters. Sci. Rep. **11**, 1-11. (10.1038/s41598-021-03343-3) 34930940 PMC8688483

[107] Wade M, Wright L, Finegold KE. 2022 The effects of early life adversity on children's mental health and cognitive functioning. Transl. Psychiatry **12**, 1-12. (10.1038/s41398-022-02001-0) 35688817 PMC9187770

[108] Palacios-Barrios EE, Hanson JL. 2019 Poverty and self-regulation: connecting psychosocial processes, neurobiology, and the risk for psychopathology. Compr. Psychiatry **90**, 52-64. (10.1016/j.comppsych.2018.12.012) 30711814

[109] Yang-Huang J, van Grieken A, You Y, Jaddoe VW, Steegers EA, Duijts L, Boelens M, Jansen W, Raat H. 2021 Changes in family poverty status and child health. Pediatrics **147**, e2020016717. (10.1542/peds.2020-016717) 33685984

[110] Roy AL, McCoy DC, Raver CC. 2014 Instability versus quality: residential mobility, neighborhood poverty, and children's self-regulation. Dev. Psychol. **50**, 1891. (10.1037/a0036984) 24842459 PMC4727396

[111] Hall PA, Fong GT. 2015 Temporal self-regulation theory: a neurobiologically informed model for physical activity behavior. Front. Hum. Neurosci. **9**, 117. 25859196 10.3389/fnhum.2015.00117 PMC4373277

[112] Prunell-Castañé A, Jurado MÁ, García-García I. 2021 Clinical binge eating, but not uncontrolled eating, is associated with differences in executive functions: evidence from meta-analytic findings. Addict. Behav. Rep. **13**, 100337. (10.1016/j.abrep.2020.100337) 33506087 PMC7815657

[113] Stevenson RJ, Francis HM. 2017 The hippocampus and the regulation of human food intake. Psychol. Bull. **143**, 1011. (10.1037/bul0000109) 28616995

[114] Braude L, Stevenson RJ. 2014 Watching television while eating increases energy intake. Examining the mechanisms in female participants. Appetite **76**, 9-16. (10.1016/j.appet.2014.01.005) 24462489

[115] Higgs S, Woodward M. 2009 Television watching during lunch increases afternoon snack intake of young women. Appetite **52**, 39-43. (10.1016/j.appet.2008.07.007) 18692103

[116] van Meer F, de Vos F, Hermans RC, Peeters PA, van Dillen LF. 2022 Daily distracted consumption patterns and their relationship with BMI. Appetite **176**, 106136. (10.1016/j.appet.2022.106136) 35697153

[117] Ritchie SJ, Bates TC, Deary IJ. 2015 Is education associated with improvements in general cognitive ability, or in specific skills? Dev. Psychol. **51**, 573. (10.1037/a0038981) 25775112 PMC4445388

[118] Barcellos SH, Carvalho LS, Turley P. 2018 Education can reduce health differences related to genetic risk of obesity. Proc. Natl Acad. Sci. USA **115**, E9765-E9772. (10.1073/pnas.1802909115) 30279179 PMC6196527

[119] Hamad R, Elser H, Tran DC, Rehkopf DH, Goodman SN. 2018 How and why studies disagree about the effects of education on health: a systematic review and meta-analysis of studies of compulsory schooling laws. Soc. Sci. Med. **212**, 168-178. (10.1016/j.socscimed.2018.07.016) 30036767 PMC6209316

[120] Schneider E, Dourish CT, Higgs S. 2022 Utility of an experimental medicine model to evaluate efficacy, side-effects and mechanism of action of novel treatments for obesity and binge-eating disorder. Appetite **176**, 106087. (10.1016/j.appet.2022.106087) 35588993

[121] Schneider E, Higgs S, Dourish CT. 2021 Lisdexamfetamine and binge-eating disorder: a systematic review and meta-analysis of the preclinical and clinical data with a focus on mechanism of drug action in treating the disorder. Eur. Neuropsychopharmacol. **53**, 49-78. (10.1016/j.euroneuro.2021.08.001) 34461386

[122] Schneider E, Martin E, Rotshtein P, Qureshi KL, Chamberlain SR, Spetter MS, Dourish CT, Higgs S. 2022 The effects of lisdexamfetamine dimesylate on eating behaviour and homeostatic, reward and cognitive processes in women with binge-eating symptoms: an experimental medicine study. Transl. Psychiatry **12**, 9. (10.1038/s41398-021-01770-4) 35013131 PMC8744047

[123] Cortese S, Moreira-Maia CR, St. Fleur D, Morcillo-Peñalver C, Rohde LA, Faraone SV. 2016 Association between ADHD and obesity: a systematic review and meta-analysis. Am. J. Psychiatry **173**, 34-43. (10.1176/appi.ajp.2015.15020266) 26315982

[124] Martin E, Dourish CT, Hook R, Chamberlain SR, Higgs S. 2020 Associations between inattention and impulsivity ADHD symptoms and disordered eating risk in a community sample of young adults. Psychol. Med. **52**, 2622-2631. (10.1017/S0033291720004638) 33272332 PMC7613803

[125] Kaisari P, Dourish CT, Rotshtein P, Higgs S. 2018 Associations between core symptoms of attention deficit hyperactivity disorder and both binge and restrictive eating. Front. Psychiatry **9**, 103. (10.3389/fpsyt.2018.00103) 29651258 PMC5884932

